# A new primer pair for barcoding of bees (Hymenoptera: Anthophila) without amplifying the orthologous *coxA* gene of *Wolbachia* bacteria

**DOI:** 10.1186/s13104-021-05845-9

**Published:** 2021-11-25

**Authors:** Christoph Bleidorn, Katharina Henze

**Affiliations:** Animal Evolution & Biodiversity, Georg-August-Universität Göttingen, Untere Karspüle 2, 37073 Göttingen, Germany

**Keywords:** Apoidea, Biomonitoring, DNA barcoding, Misamplification

## Abstract

**Objectives:**

DNA barcoding became an effective method for the identification and monitoring of bees. However, standard primer pairs used for barcoding often result in (co-) amplification of bacterial endosymbionts of the genus *Wolbachia*, which are widespread among bee species. Here we designed a new primer pair and compared it with the performance of the standard Folmer-primers for a small sample set of bees representing the main taxonomic groups of bees.

**Results:**

The newly designed primer pair (BeeCox1F1/BeeCox1R2) outperformed the standard barcoding primer (LCO1490/HCO2198). By generating barcodes for a small test set of bees we found that the new primer pair produced high-quality sequences in all cases for unambiguous species identification using BOLD. Conversely, the standard barcoding primers often co-amplified the homologous *Wolbachia* gene and resulted in mixed chromatogram signals. These sequences showed high similarity with the bacterial endosymbiont instead of the host.

## Introduction

More than 20,000 species of bees (Hymenoptera, Anthophila) are estimated to occur worldwide [1] and they became a posterchild for conservation biology as they play a vital role in pollination in both natural and managed ecosystems [2]. Unfortunately, bee diversity and abundance has been reported to decline at different levels across continents [3]. Extensive faunistic inventories are necessary to better understand changes in occurrence of bee species across scales. However, bee taxonomy can be difficult as exhaustive identification keys are available for only some taxonomic groups and few geographic regions. Moreover, sometimes confident identification is possible only in one of the sexes (e.g., *Andrena ovatula* group), and (nearly) cryptic species complexes have been described in several recent revisions of selected taxa [4]. Different ways to accelerate bee identification and biomonitoring have been suggested [5, 6]. The most prominent approach is DNA barcoding, where a specific segment of the mitochondrial cytochrome *c* oxidase 1 gene (*cox1*) is used for species identification [7]. As such, several geographic region specific barcoding initiatives for bees have been launched or already successfully finished, e.g., Central Europe [8], Ireland [9], Canada [10], providing the necessary background for DNA-based identification.

**Fig. 1:**
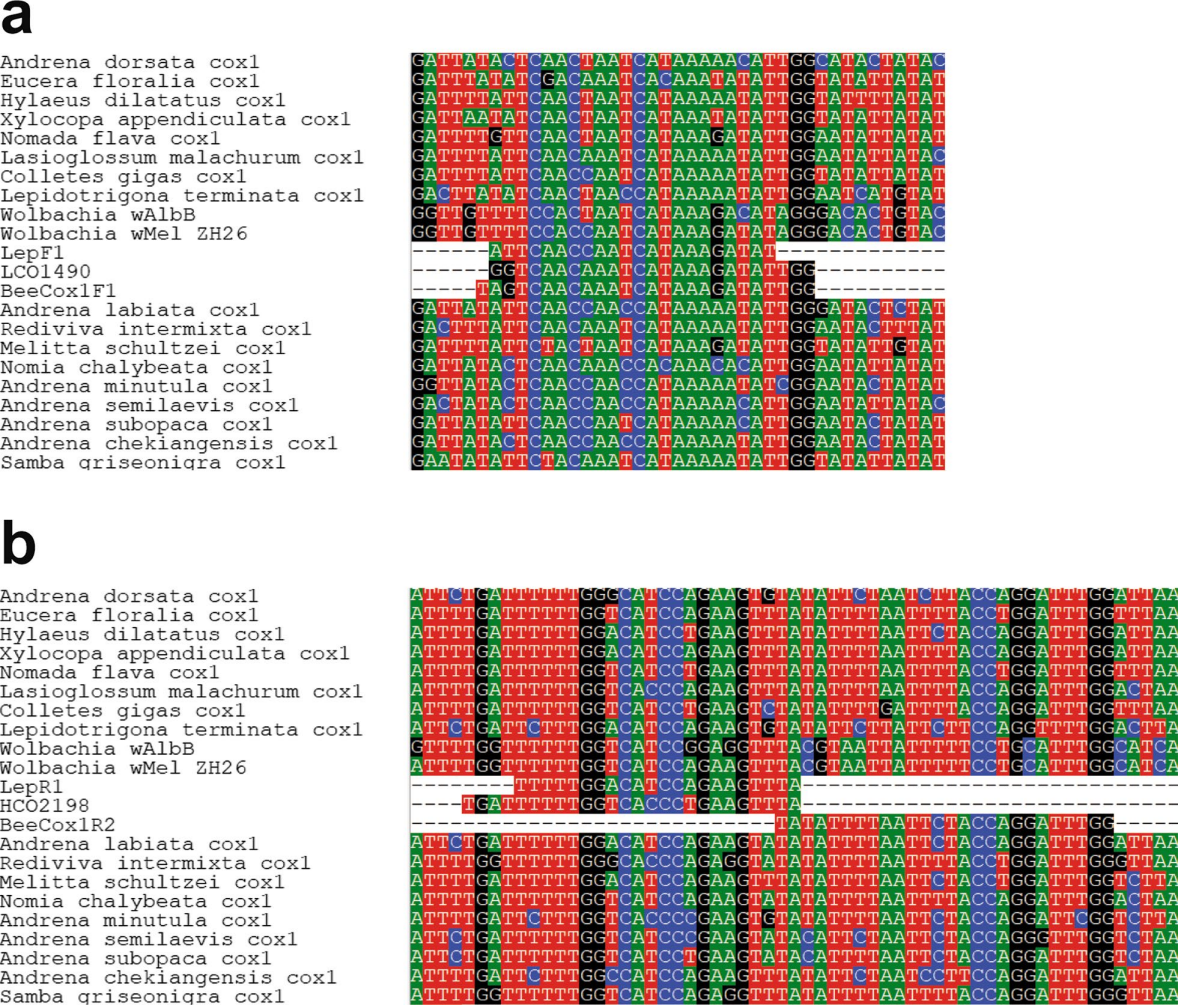
Multiple sequence alignment. Snap shot of the multiple alignment of bee *cox1*-sequences (and two sequences of the homologous *Wolbachia* gene) indicating the position of the forward (**a**) and reverse (**b**) primer sequences of the standard (LCO1498/HCO2198, LepF1/LepR1) and new (BeeCox1F1/BeeCox1R2) primer pairs

Primarily, the success of DNA barcoding depends on the specificity of the primers to the target organism’s *cox1* gene. Intracellular Alphaproteobacteria of the genus *Wolbachia* have been recorded from (terrestrial) arthropods and selected nematodes [11]. Presence of these bacteria often causes co-amplification of the orthologous *coxA* gene along with (or instead of) the host *cox1* gene. More than 60% of the native German bee species have been reported to be infected by *Wolbachia* [12, 13]. Several previous studies reported that the standard DNA barcoding primers (e.g., LCO1490 and HCO2198 [14], LepF1 and LeR1 [15]) often resulted in mixed amplicons and poor-quality sequences in bees [9, 16]. Further, screening of the BOLD database revealed the highest number of unintended amplifications of *Wolbachia* DNA in Hymenoptera [13]. There seem to be taxon-specific patterns regarding the frequency of infected species, and especially a high number of species from the species-rich genera *Andrena*, *Halictus*, *Lasioglossum*, *Nomada* or *Sphecodes* are infected [13]. In congruence with the reports by [9], our routine work in the lab showed that individuals of these genera are difficult to barcode using standard approaches, as sequencing revealed mixed signals or the *Wolbachia* sequence. This comes to no surprise, as the most commonly used standard barcoding primers (LCO1490/HCO2198, LepF1/LepR1) actually show a high similarity to the homologous region of the *Wolbachia coxA* gene (Fig. 1). Though alternative primer pairs have been already suggested as a workaround, their annealing temperature seems relatively low (and therefore unspecific) [17] or consist of highly degenerative priming sites [16]. These features could hamper the sequencing and result in poor sequences. Here we present a newly designed primer combination and test its suitability for a phylogenetically representative taxon sampling of bees, with a special focus on its suitability in the case of *Wolbachia* infected species.

## Main text

### Methods

#### Species collection and DNA extraction

A total of 48 bees, representing 44 species and 18 genera, were collected from sand and gravel pits located in the FFH-protected area “Ballertasche” (51° 27′ 30.3ʺ N 9° 38′ 10.2ʺ E) and the nature-sanctuary “NSG Stadtwald Göttingen und Kerstlingeröder Feld” (51° 32′ 36.1ʺ N 10° 01′ 58.7ʺ E). The specimens were identified based on morphology [18–25] to the genus or species level and later processed for molecular lab work. Thorax muscle tissue was removed using forceps and transferred directly into an Eppendorf cap for DNA extraction using the Qiagen DNEasy Blood and Tissue Kit (Qiagen, Hilden).

#### Primer design and PCR

Initially, selected bees including difficult to identify groups of species (e.g., females of the *Andrena ovatula* group) were used for DNA barcoding using the standard Folmer primers (LCO1490/HCO2198). However, in many cases the resultant sequences were either from *Wolbachia* endosymbionts or had background noise with multiple signals (see below for details on results). Thus, a new set of primers were designed by downloading the complete *cox1* sequences (around 1500 bp in length) of phylogenetically representative species of bees (n = 66) and, the orthologous gene region of *Wolbachia* sp. from the NCBI Genbank.

The sequences were designed using the MAFFT online tool [26] by keeping default parameters and automatic strategy selection for alignment. Further, the traditional barcoding primers used for insects (LCO1490/HCO2198, LepF1/LepR1) were also aligned with the sequences to delimit the barcoding region within the gene. After alignment, the primers were designed in such a way that the reverse primer binding site is divergent from *Wolbachia* species and cannot amplify the orthologous coxA gene. The alignment including NCBI accession numbers is available at https://github.com/Animal-Evolution-and-Biodiversity/Design_Barcoding_Primer_Bees/tree/main/Alignment.

The newly designed primer pair was named BeeCox1F1 (TAGTCAACAAATCATAAAGATATTGG) and BeeCox1R2 (CCAAATCCTGGTAGAATTAAAATATA). The expected PCR amplicon size is around 670 bp, a bit larger than that of the standard barcoding primer pairs (~ 650 bp). We used the NetPrimer online tool (http://www.premierbiosoft.com/netprimer/) to check the suitability of this primer pair (melting temperature, secondary structures and cross dimers) and validated by amplifying the *cox1* gene from the bees that have shown misamplification with the standard primer pairs (Table 1).

**Table 1:** Species level taxonomic assignment success^a^ of barcodes from different primer pairs of selected bee species representing different families

Taxon	LCO1490/HCO2198	BeeCox1F1/BeeCox1R2
Megachilidae		
* Anthidium punctatum* (107)^b^	CORRECT	CORRECT
* Chelostoma florisomne* (99)	CORRECT	CORRECT
* Coelioxys alata* (1)	CORRECT	CORRECT
* Coelioxys elongata* (110)	CORRECT	CORRECT
* Coelioxys rufescens* (108)	CORRECT	CORRECT
* Herides truncorum* (112)	CORRECT	CORRECT
* Osmia bicolor* (93)	CORRECT	CORRECT
* Megachile circumcincta (2)*	CORRECT	CORRECT
* Megachile willughbiella (104)*	CORRECT	CORRECT
* Megachile versicolor* (95)	CORRECT	CORRECT
Andrenidae		
* Andrena barbilabris* (4)	CORRECT	CORRECT
* Andrena flavipes (6)*	WOLBACHIA	CORRECT
* Andrena fulvago (105)*	MIXED	CORRECT
* Andrena ovatula* (5)	MIXED	CORRECT
* Andrena ovatula* (7)	WOLBACHIA	CORRECT
* Andrena minutula* (15)	CORRECT	CORRECT
* Andrena nigroaenea* (8)	WOLBACHIA	CORRECT
* Andrena wilkella* (35)	MIXED	CORRECT
* Andrena wilkella* (36)	MIXED	CORRECT
* Andrena wilkella* (37)	MIXED	CORRECT
Halictidae		
* Lasioglossum minutulum* (14)	MIXED	CORRECT
* Lasioglossum nitidiusculum* (41)	CORRECT	CORRECT
* Lasioglossum pauxillum* (49)	CORRECT	CORRECT
* Lasioglossum semilucens* (46)	WOLBACHIA	CORRECT
* Lasioglossum sexstrigatum* (16)	CORRECT	CORRECT
* Lasioglossum villosulum* (48)	CORRECT	CORRECT
* Sphecodes ferruginatus* (97)	CORRECT	CORRECT
* Sphecodes miniatus* (19)	CORRECT	CORRECT
* Sphecodes monilicornis* (18)	WOLBACHIA	CORRECT
* Sphecodes puncticeps* (17)	CORRECT	CORRECT
Colletidae		
* Colletes daviesanus* (117)	WOLBACHIA	CORRECT
* Hylaeus brevicornis* (13)	CORRECT	CORRECT
* Hylaeus communis* (12)	WOLBACHIA	CORRECT
* Hylaeus confusus* (106)	CORRECT	CORRECT
Apidae		
* Anthophora furcata* (102)	CORRECT	CORRECT
* Bombus pratorum* (101)	CORRECT	CORRECT
* Bombus rupestris* (91)	CORRECT	CORRECT
* Bombus terrestris* (72)	CORRECT	CORRECT
* Ceratina cyanea* (113)	MIXED	CORRECT
* Epeolus variegatus* (115)	CORRECT	CORRECT
* Eucera nigrescens* (114)	CORRECT	CORRECT
* Nomada fabriciana* (96)	CORRECT	CORRECT
* Nomada goodeniana* (92)	CORRECT	CORRECT
* Nomada marshamella* (98)	CORRECT	CORRECT
* Nomada ruficornis* (118)	MIXED	CORRECT
* Nomada succincta* (71)	MIXED	CORRECT
Mellitidae		
* Melitta haemorrhoidales* (109)	CORRECT	CORRECT
* Melitta leporina* (M10)	MIXED	CORRECT

^a^Assignment success was either given as unambiguous (correct) identification in BOLD as CORRECT, misamplification of endosymbiont as WOLBACHIA, or a sequence that showed double or mixed signal and could not be identified as MIXED

^b^Number in parentheses correspond to unique identifiers with the online available sequence chromatograms and data

In this study, we compared the efficiency of the new BeeCox1F1/BeeCox1R2 primer combination with the standard LCO1490/HCO2198 primer. The PCR regime for the new primer, BeeCox1F1/BeeCox1R2 was as follows: Initial denaturation at 94 °C for 2 min; 40 cycles of 30 s at 94 °C, 45 s at 50 °C and 1 min at 72 °C; Final extension for 10 min at 72 °C. For the LCO1490/HCO2198 primer, PCR conditions were as follows: Initial denaturation at 94 °C for 2 min; 40 cycles of 30 s at 94 °C, 45 s at 46 °C and 1 min at 72 °C; Final extension for 10 min at 72 °C. All PCRs were carried out in a total volume of 25 µl, containing 1 µl of each primer (10 pM), 12.5 µl DreamTaq Green PCR Master Mix (Thermo Fisher), 1 µl of genomic DNA and 9.5 µl of ddH2O. Negative controls containing water instead of template DNA were included in all PCRs. Sanger sequencing and basecalling (> QV20 using the KB basecaller v3.1) for forward directions for all fragments was performed by Microsynth Seqlab GmbH (Germany). All chromatograms and base-called fasta-files from Sanger sequencing are available at https://github.com/Animal-Evolution-and-Biodiversity/Design_Barcoding_Primer_Bees/tree/main/Sanger_sequence_files.

#### Taxonomic assignment

Sequence quality was verified by inspecting the chromatograms using Chromas 2.6.6 (http://technelysium.com.au/wp/chromas/). The sequences were assigned to the species by subjecting them similarity analysis with the BOLD reference database. The similarity value within a range of 98–100% was considered as the threshold value for taxonomic assignment. The results were categorized as (i) unambiguous identification with the barcode sequence, (ii) ambiguous identification showing similarity with *Wolbachia CoxA* sequence and (iii) no match with reference sequences due to poor sequence quality.

### Results and discussion

#### PCR and sequencing results

We were able to produce PCR amplification products for both primer pairs for all 48 bee individuals included in this study, altogether resulting in 96 PCR products. Our taxon sampling hereby represents six of the seven described families of Anthophila. We focussed on members of Andrenidae and Halictidae, as they had shown before to be more difficult to be barcoded (see above), however for all available bee families we included several representatives to study the performance of the primer pairs. After sequencing, we found 8 sequences with a mixed signal, while 88 sequences could be used for identification using the BOLD database. Sequences with mixed signals were all amplicons from the LCO1490/HCO2198 primer pair, and in four cases stem from individuals belonging to the *Andrena ovatula*-group.

#### Taxonomic assignment

Altogether 88 sequences were identified using the BOLD database. None of these sequences showed frame-shifts or stop codons, which suggest that possible NUMTs were not amplified. In the case of 66 barcodes from 33 individuals both primer pairs produced the same result and the corresponding taxonomic assignment of the bee species was unambiguous. In the case of seven individuals, the sequence from the LCO1490/HCO2198 primer pair was taxonomically assigned to originate from a *Wolbachia* endosymbiont, while the corresponding barcodes from the BeeCox1F1/BeeCox1R2 primer pair allowed an unambiguous taxonomic assignment to a bee species. The same is true for the 8 barcodes which could only be analysed for the BeeCox1F1/BeeCox1R2 primer pair, as the LCO1490/HCO2198 primer pair resulted in a mixed signal of the sequenced barcode.

### Conclusion

By comparing the location of the standard barcoding primer pairs in the multiple sequence alignment of complete *cox1*-sequences of bees and recognizing its similarity with the corresponding *Wolbachia* gene sequences, we designed a new primer pair circumventing these matches (Fig. 1). We were able to produce PCR amplification products for the new primer pair and the standard Folmer-primers for all 48 bee individuals included in our small test sample set, representing six of the seven higher taxa (“families”) recognized within Anthophila. Even among this small sample size we found seven cases where the Folmer-primers amplified *Wolbachia* instead of the host cox-1 gene and eight cases of mixed signal, which might also stem from simultaneous amplification of host and endosymbiont DNA. In contrast, with the newly designed BeeCox1F1/ BeeCox1R2-primer pair we were in all cases able to generate clear sequences which could be identified as the corresponding bee species in the BOLD database. As such the newly proposed primer pair should help to reduce problems when barcoding bees, which will be especially important for morphologically difficult to identify species, such as from the *Andrena ovatula* group.

## Limitations

The new barcoding primer pair has been tested for a phylogenetically representative, but small taxon sampling of bees. It is also unclear how they will perform when barcoding related taxa of Hymenoptera. Whereas we are convinced that this barcoding primer pair will circumvent problems related to mis-amplification of endosymbionts of the genus *Wolbachia*, possible amplification of nuclear integrations of mitochondrial sequences (NUMTs) remains an issue for DNA barcoding [27].

## Data Availability

Original chromatograms and sequence data for all barcoded bees in this study, as well as the multiple alignment for primer design can be found at https://github.com/Animal-Evolution-and-Biodiversity/Design_Barcoding_Primer_Bees.

## Abbreviations

BOLD: Barcode of Life Data System
PCR: Polymerase chain reaction
